# Analysis of the Dynamic Sensitivity of Hemisphere-Shaped Electrostatic Sensors’ Circular Array for Charged Particle Monitoring

**DOI:** 10.3390/s16091403

**Published:** 2016-08-31

**Authors:** Xin Tang, Zhong-Sheng Chen, Yue Li, Zheng Hu, Yong-Min Yang

**Affiliations:** Science and Technology on Integrated Logistics Support Laboratory, National University of Defense Technology, Changsha 410073, China; tangxin11_nudt@163.com (X.T.); liyue@nudt.edu.cn (Y.L.); zhenghu@nudt.edu.cn (Z.H.); yangyongmin@163.com (Y.-M.Y.)

**Keywords:** electrostatic sensor array, dynamic sensitivity, hemisphere-shaped electrostatic sensor, circular array, array signal processing algorithm

## Abstract

Electrostatic sensor arrays (ESAs) are promising in industrial applications related to charged particle monitoring. Sensitivity is a fundamental and commonly-used sensing characteristic of an ESA. However, the usually used spatial sensitivity, which is called static sensitivity here, is not proper for moving particles or capable of reflecting array signal processing algorithms integrated in an ESA. Besides, reports on ESAs for intermittent particles are scarce yet, especially lacking suitable array signal processing algorithms. To solve the problems, the dynamic sensitivity of ESA is proposed, and a hemisphere-shaped electrostatic sensors’ circular array (HSESCA) along with its application in intermittent particle monitoring are taken as an example. In detail, a sensing model of the HSESCA is built. On this basis, its array signals are analyzed; the dynamic sensitivity is thereupon defined by analyzing the processing of the array signals. Besides, a component extraction-based array signal processing algorithm for intermittent particles is proposed, and the corresponding dynamic sensitivity is analyzed quantitatively. Moreover, simulated and experimental results are discussed, which validate the accuracy of the models and the effectiveness of the relevant approaches. The proposed dynamic sensitivity of ESA, as well as the array signal processing algorithm are expected to provide references in modeling, designing and using ESAs.

## 1. Introduction

Electrostatic monitoring systems (EMSs) are featured with the advantages of robustness and low cost [1,2], making them very promising in parameter monitoring of gas-solid flows [3,4,5], exhaust debris monitoring-based PHM of gas turbines [1,6,7], etc. Electrostatic sensors are key components of EMSs. Among them, hemisphere-shaped electrostatic sensors were proposed by us as a trade-off between intrusive and non-intrusive electrostatic sensors. They combine the advantages of relatively high sensitivity, easy installation, flexible layout, accuracy in theoretical modeling and cause very little disturbance to flows [8,9].

The sensing principle of an electrostatic sensor is electrostatic induction. It determines that even if a particle carries a constant charge, the corresponding induced charge on an electrostatic sensor probe will reduce sharply when the distance between the particle and the probe gets larger. As a result, a near particle with quite weak charge can generate similar signals as a far particle with great charge. That is to say, the charge quantity of a particle can hardly be accurately monitored by a single electrostatic sensor if the position of the particle is not provided. In view of the sensitivity, this means that most electrostatic sensors have the drawback of inhomogeneous and quite localized sensitivity; thus, only particles near the probe can be effectively detected [8,10]. In a word, as for the applications that need the accurate particle charge (e.g., particle size monitoring or gas-solid flow density monitoring), an EMS that contains a single electrostatic sensor is usually of poor monitoring accuracy. Moreover, a total failure of the whole system will occur if only the sensor breaks down. Therefore, in order to obtain better monitoring accuracy and reliability, EMSs composed of multiple electrostatic sensors have become the trend [5,11,12]. They are usually called electrostatic sensor arrays (ESAs), and the electrostatic sensors are called the sensor unit. Two aspects are of high concern in relevant research: the first is the hardware design and related sensing characteristics analysis; the second is the application and related array signal processing algorithms.

Circular ESA is a kind of representative hardware structure, which is made up by placing some identical electrostatic sensors uniformly around a circular pipeline in one cross-section [12,13,14,15,16,17,18]. Xu et al. used arc-sharped electrostatic sensors to construct circular ESAs and used the finite element method to study the sensing characteristics of the sensor units. Some geometric and material influence factors on the sensing characteristics were also analyzed [18]. Thuku et al. studied circular ESAs composed of rod-shaped electrostatic sensors [5,19]. A sensing model of the sensor units was built, and a comparison between four and 16 sensor systems was made [17]. In addition, differently-sized circularplate-shaped and rectangular plate-shaped electrostatic sensors were modeled and analyzed [20]; they are also suitable for circular ESAs. However, existing works on the sensing characteristics were generally focused on individual electrostatic sensors, thus lacking descriptions and models for the sensing characteristics of a whole ESA. In addition, as a fundamental and the most commonly-used sensing characteristic, sensitivity should reflect the monitoring accuracy of an EMS directly. As for an ESA, this means that a proper sensitivity definition should build a direct relationship between the monitored particles and the monitoring parameters of the whole ESA. However, the usually used spatial sensitivity only builds a basic relationship between static charged particles and corresponding induced charge on a sensor probe [10,18,21]; thus, it is called static sensitivity here. In practice, particles to be monitored are always moving. Furthermore, as a three-dimensionally-defined parameter, static sensitivity is difficult to accurately model when real boundary conditions are considered [9]. More importantly, follow-up processing of the induced charge is not considered in the definition, such as charge-voltage transformation and array signal processing algorithms. In a word, in order to build a direct relationship between the monitored particles and the monitoring parameters of a whole ESA, thus reflecting its monitoring accuracy directly, a more practical sensitivity definition needs to be defined in a systemic perspective.

In the aspect of application, a circular ESA is usually used to detect the particle distribution over the cross-section of a pipeline and further infer gas-solid flow parameters, such as particle density and flow regime. In these cases, the particles to be monitored are continuous in the time domain. That is to say, there are always numerous particles passing a cross-section simultaneously, thus forming an obvious profile of the solid-phase, which can be imaged by tomography-based methods [5,11,14]. ESAs with arc-shaped sensor units usually adopt array signal processing algorithms based on electrical capacitance tomography [11,12,16,22,23]. They are suitable for dense gas-solid flows. There are also reports on volume tomography using multi-circular ESAs with arc-shaped sensor units [24,25,26]. ESAs with rod-shaped sensor units usually adopt algorithms based on electrostatic tomography [5,14,18]. They are suitable for both dense and dilute gas-solid flows. However, because of the limited number of sensor units, the tomography-based algorithms are faced with tough ill-conditioned problems, which lead to poor image resolution [11,16]. Improvements can be made by increasing the unit number [17] and adopting some iterative regularization methods, filtering methods, error minimization protocols, etc., which will increase the computation time [11,12,16]. As a result, the tomography-based algorithms only indicate approximate areas where particles concentrate, but they cannot accurately confirm where discrete particles are located. Moreover, the algorithms are more suitable for relatively stable flows, because the monitoring accuracy can be improved by making a compromise on the computation time. In contrast, particles to be monitored could be quite intermittent in some applications. That is to say, there are only one or just a few particles passing a cross-section simultaneously. One representative application is exhaust debris monitoring-based PHM of gas turbines [7,17,18,19,20,21,22,23,24,25,26,27,28,29]. In this case, the occurrences of fault-related particles are accidental and instantaneous. In other words, first, the particles to be monitored are quite intermittent. Second, a fast array signal processing algorithm is needed to avoid missing the fast-passing particles. Besides, the size and charge quantity of the particles, rather than the distribution, are concerned. A measurement technique was studied by Addabbo et al. to estimate the trajectory position, charge and velocity of a moving charged particle. It was based on a small array whose electrostatic sensors have improved low-frequency response [30,31]. However, relevant studies are scarce yet. As for a circular ESA, array signal processing algorithms remain to be designed for the intermittent particle monitoring.

To solve the problems above, the dynamic sensitivity of ESA is proposed to describe the sensitivity of ESAs in a systemic perspective. An ESA called the hemisphere-shaped electrostatic sensors’ circular array (HSESCA) along with its application in intermittent particle monitoring are taken as an example. In detail, a sensing model of the HSESCA, as well as that of the sensor units is built in Section 2. On this basis, array signals of the HSESCA are analyzed, and the dynamic sensitivity of ESA is thereupon defined in Section 3. A component extraction-based array signal processing algorithm for discrete particle monitoring is designed in Section 4. Numerical simulations and experiments are done for validations in Section 5. The main results are concluded in Section 6.

## 2. Sensing Model of an HSESCA

This section builds a quantitative relationship between a charged particle and the induced charge of an HSESCA, which provides a theoretical foundation for this paper.

### 2.1. Basic Structure of an HSESCA

**Figure 1:**
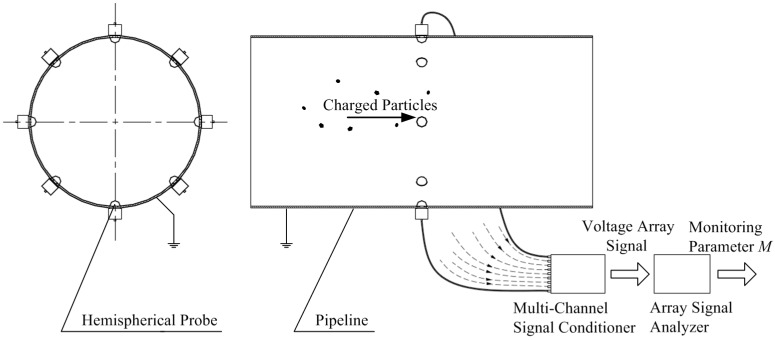
Schematic of a grounded pipeline-installed HSESCA.

An HSESCA with eight sensor units is installed on a grounded pipeline, as shown in Figure 1. It is mainly composed of eight identical hemispherical probes, a multi-channel signal conditioner and an array signal analyzer. Compared to a “flat head” probe leaning inward toward the pipeline, it is much easier to build a theoretical sensing model for a hemispherical probe, because it only contains a simple hemispheric surface. In addition, a hemispherical probe should be more durable than a “flat head” probe under severe working conditions, as a hemispheric surface is free of damageable edges.

**Figure 2:**
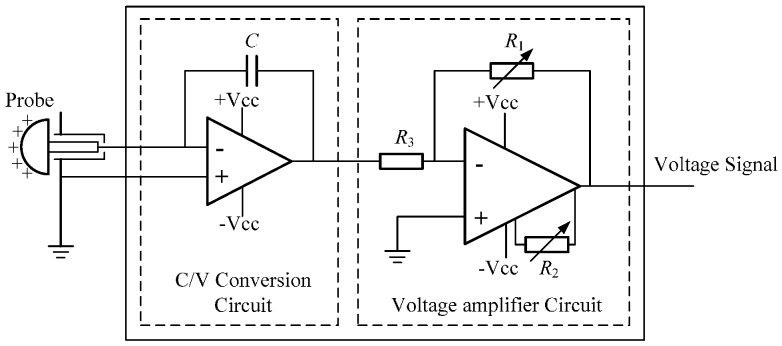
Schematic of a proportional two-stage signal conditioner channel.

The hemispherical probes are uniformly installed in a cross-section around the pipeline. It is called the observation cross-section. Once the charged particles pass, the induced charge will be generated on each probe and then converted into voltage array signals immediately by the multi-channel signal conditioner for post-processing. Every channel of the conditioner is designed as an identical proportional two-stage amplifier as in Figure 2 [9]. The induced charge is proportionally transformed into voltage in the first stage and then negatively amplified in the second stage. Therefore, the total function of each channel is represented by a negative amplification coefficient *K* whose unit is V/C. A hemispherical probe and its signal conditioner channel compose an independent sensor unit of the HSESCA. The array signal analyzer is composed of a signal acquisition card and a computer. Some specially-designed array signal processing algorithms are usually integrated in the computer to calculate the monitoring parameters (denoted as *M*) of the whole HSESCA.

As the sensitivity characteristics of an electrostatic sensor are quite localized [8,9,10] and the radius of the probes is much smaller than the distances between them, interactions between the probes can be ignored. Thus, a sensor unit is firstly modeled to lay a foundation for the whole HSESCA.

### 2.2. Theoretical Modeling of a Hemisphere-Shaped Electrostatic Sensor Unit

**Figure 3:**
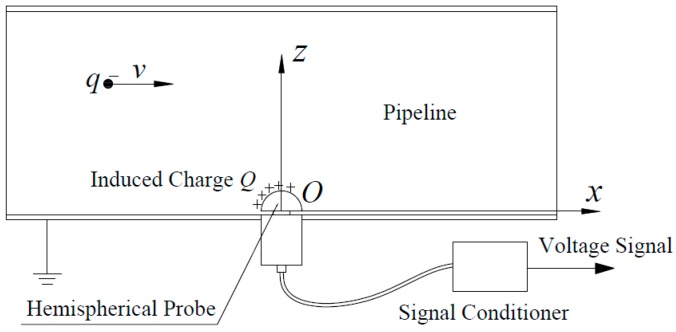
Schematic of a grounded pipeline-installed sensor unit of the HSESCA.

As shown in Figure 3, one sensor unit is kept. A Descartes coordinate system is built with the center of the probe’s bottom face set as the origin. The axial direction of the pipeline and that of the probe are set as the *x*-axis and the *z*-axis, respectively. Then, the *y*-axis is determined by the right-hand rule automatically. Accordingly, a point in the pipeline is denoted as *P*(*x*, *y*, *z*).

Because electrostatic equilibrium states are reached instantaneously (10^−19^ s), the interaction between the probe and charged particles in the pipeline can be described by a pure electrostatic field [18,21]. It is uniquely determined by the Poisson equation and Dirichlet boundary conditions as:

(1) $$\nabla^{2}\varphi_{t}\left(P\right)=-\frac{\rho_{t}\left(P\right)}{\varepsilon },s.t.\left\{ \begin{array}{l} \varphi_{t}\left(P\right)=0,P\in \Gamma_{2}\\ \varphi_{t}\left(P\right)=\mathrm{const}\left(t\right),P\in \Gamma_{1}\\ \underset{P\to \infty }{\lim}\varphi_{t}\left(P\right)=0 \end{array}\right.$$

where Γ_1_ is the hemispherical surface of the probe and Γ_2_ is the inner wall of the pipeline. *ρ_t_*(*P*) is the volume charge density in the pipeline at time *t*, and *φ_t_*(*P*) is the corresponding potential distribution. Besides, const(*t*) is a constant function of *t*, describing the equipotential surface of the probe. ε is the permittivity of free space, and $\nabla^{2}$ is the Laplace operator.

On account of the pipeline being much bigger than the probe, the electrostatic field on the inner wall is simplified as that on one infinite plane [2,6,17]. In this case, when a charged particle is simplified as a point charge *q* [8,10], *φ_t_*(*P*) is firstly solved using the Green function and the method of image charges. Then, the sensing model at *P*(*x*, *y*, *z*) is obtained according to the relationship between the induced charge and the potential distribution on a conductor surface [8,9]. That is:

(2) $$\begin{array}{l} Q\left(x,y,z\right)=-\frac{qa}{b}-\frac{qz}{2\pi }\int_{0}^{2\pi }d\theta \int_{0}^{a}{\left(r^{2}+b^{2}-2xy\cos\theta -2yr\sin\theta \right)}^{-\frac{3}{2}}rdr\\ \;+\frac{qa^{3}z}{2\pi b^{3}}\int_{0}^{2\pi }d\theta \int_{0}^{a}{\left[r^{2}+\frac{a^{4}-2a^{2}\left(xr\cos\theta +yr\sin\theta \right)}{b^{2}}\right]}^{-\frac{3}{2}}rdr \end{array}$$

where *Q* is the total induced charge on the probe, *a* is the radius of the probe and $b=\sqrt{x^{2}+y^{2}+z^{2}}$ is the distance between the point charge and the origin.

### 2.3. Theoretical Modeling of an HSESCA

**Figure 4:**
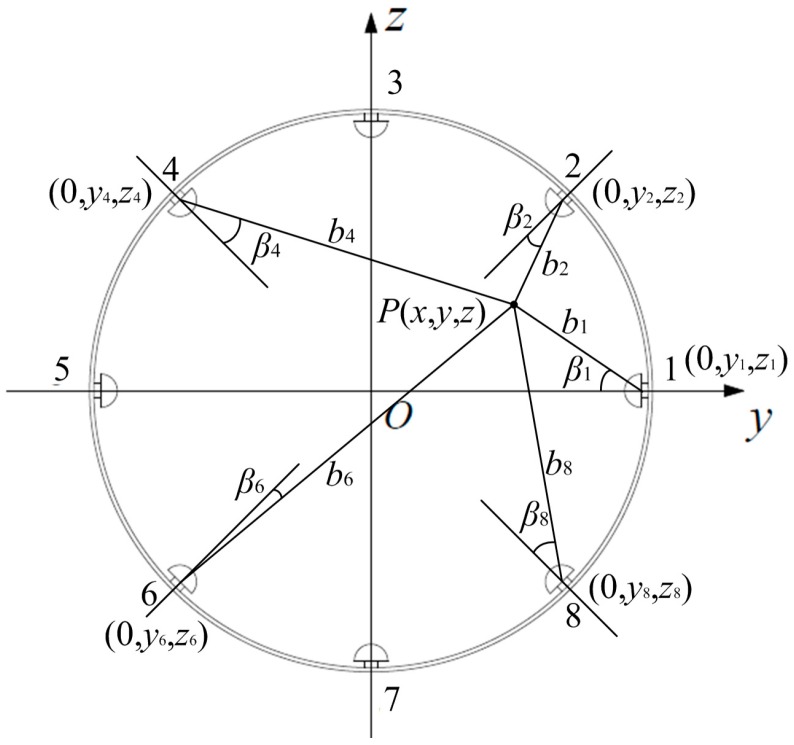
Schematic of *b_i_* and *β_i_* in the new Descartes coordinate system.

In order to build a sensing model for all of the sensor units in the same coordinate system, firstly, according to the symmetry of the probe, Equation (2) is transformed into:

(3) $$\begin{array}{l} Q\left(x,y,z\right)=-\frac{qa}{b}-\frac{qb\cos\beta }{2\pi }\int_{0}^{2\pi }d\theta \int_{0}^{a}{\left(r^{2}+b^{2}-2br\sin\beta \sin\theta \right)}^{-\frac{3}{2}}rdr\\ \;+\frac{qa^{3}\cos\beta }{2\pi b^{2}}\int_{0}^{2\pi }d\theta \int_{0}^{a}{\left(r^{2}+\frac{a^{4}}{b^{2}}-\frac{2a^{2}r\sin\beta \sin\theta }{b}\right)}^{-\frac{3}{2}}rdr \end{array}$$

where β=arccos(z/b) is the included angle between the line from the point charge to the origin and the axis of the probe. Then, a new Descartes coordinate system is built by translating the original one (see Figure 2) to the center of the observation cross-section. Meanwhile, the sensor units are numbered anticlockwise, with coordinates of the *i*-th probe denoted as (0, *y_i_*, *z_i_*) and those of the point charge denoted as *P*(*x*, *y*, *z*) in the new coordinate system; as shown in Figure 4.

Thereupon, the sensing model of the *i*-th sensor unit is derived from Equation (3) as:

(4) $$\begin{array}{l} Q_{i}\left(x,y,z\right)=-\frac{qa}{b_{i}}-\frac{qb_{i}\cos\beta_{i}}{2\pi }\int_{0}^{2\pi }d\theta \int_{0}^{a}{\left(r^{2}+{b_{i}}^{2}-2b_{i}r\sin\beta_{i}\sin\theta \right)}^{-\frac{3}{2}}rdr\\ \;+\frac{qa^{3}\cos\beta_{i}}{2\pi {b_{i}}^{2}}\int_{0}^{2\pi }d\theta \int_{0}^{a}{\left(r^{2}+\frac{a^{4}}{{b_{i}}^{2}}-\frac{2{a^{2}}_{i}r\sin\beta_{i}\sin\theta }{b_{i}}\right)}^{-\frac{3}{2}}rdr \end{array}$$

where *b_i_* is the distance from the charged particle to the *i*-th probe and *β_i_* is the included angle between the point charge and the axis of the *i*-th probe. They are determined by geometrical relationships:

(5) $$b_{i}=\sqrt{x^{2}+{\left(y-y_{i}\right)}^{2}+{\left(z-z_{i}\right)}^{2}}$$

(6) $$\beta_{i}=\arccos\left[({y_{i}}^{2}+{z_{i}}^{2}-yy_{i}-zz_{i})/lb_{i}\right]$$

where $l=\sqrt{{y_{i}}^{2}+{z_{i}}^{2}}$ is the distance between the origin *O* and the bottom face of a probe.

Further, the sensing model of the HSESCA is obtained by making a superposition of sensing models of all of the sensor units. That is:

(7) $$\begin{array}{c} Q_{A}\left(x,y,z\right)=\sum_{i}Q_{i}\left(x,y,z\right)=-\sum_{i}\frac{qa}{b_{i}}-\sum_{i}\frac{{qb}_{i}\cos\beta_{i}}{2\pi }\int_{0}^{2\pi }d\theta \int_{0}^{a}{\left(r^{2}+{b_{i}}^{2}-2b_{i}r\sin\beta_{i}\sin\theta \right)}^{-\frac{3}{2}}rdr\\ \;+\sum_{i}\frac{qa^{3}\cos\beta_{i}}{2\pi {b_{i}}^{2}}\int_{0}^{2\pi }d\theta \int_{0}^{a}{\left(r^{2}+\frac{a^{4}}{{b_{i}}^{2}}-\frac{2{a^{2}}_{i}r\sin\beta_{i}\sin\theta }{b_{i}}\right)}^{-\frac{3}{2}}rdr \end{array}$$

In addition, an FEM-based calibration method was proposed as a supplement to consider the actual boundary conditions [9]. It is used to improve the accuracy of the sensing models when a charged particle is in the observation cross-section. The main steps are as follows: First, an FEM model of a grounded pipeline-installed probe is built. The model provides simulated induced charge with high fidelity. Then, the relative error between the simulated and the theoretical induced charge is analyzed. On this basis, a calibration item calculated by using fitting methods is added to eliminate the relative error. When the inner radius of the pipeline is 200 mm and the button face of a probe is 2 mm apart away from the inner wall, Equation (4) is calibrated as:

(8) $$Q_{i,x=0}^{\prime }\left(y,z\right)=\left\{ \begin{array}{ll} Q_{i,x=0}\left(y,z\right)\left(p_{1}{b_{i}}^{p_{2}}+p_{3}\;\right) & ,\;\mathrm{if}\;b_{i}\geq D\\ Q_{i,x=0}\left(y,z\right) & ,\;\mathrm{if}\;b_{i}<D \end{array}\right.$$

where *D* = −0.0188 *β_i_* + 0.0444 is the calibration boundary that is determined according to the variation trend of the relative error [10]. In the area of *b_i_* < *D*, the relative error is negligible; thus, the sensing model is only calibrated where *b_i_* ≥ *D*. The calibration parameters are obtained using fitting methods; they are:

(9) $$\left\{ \begin{array}{l} p_{1}=-5.7311\times 10^{-8}e^{8.7639\beta_{i}^{2}}-6.103e^{0.5724\beta_{i}^{2}}\\ p_{2}=-43.9996e^{0.1261\beta_{i}^{2}}+45.9113e^{0.113\beta_{i}^{2}}\\ p_{3}=1.0479e^{7.0643\times 10^{-2}\beta_{i}^{2}}+1.2218\times 10^{-4}e^{4.4059\beta_{i}^{2}} \end{array}\right.$$

It is obvious that Equation (7) is calibrated as $Q_{A,x=0}^{\prime }\left(y,z\right)=\sum_{i}Q_{i,x=0}^{\prime }\left(y,z\right)$.

## 3. Definition of the Dynamic Sensitivity of ESA

### 3.1. Definition of the Static Sensitivity of ESA

In order to make a comparison with the dynamic sensitivity of ESA, the static sensitivity of ESA is defined here. The static sensitivity of an electrostatic sensor is defined as the absolute induced charge on the probe generated by a unit charge [7,8,21]. Analogously, the static sensitivity of ESA is defined as the absolute induced charge on all of the probes of an ESA generated by a unit charge. That is:

(10) $$S_{A}\left(x,y,z\right)=-\frac{Q_{A}\left(x,y,z\right)}{q}$$

The minus indicates that the induced charge and the point charge *q* are of opposite signs. As for an HSESCA, its static sensitivity model is derived from Equations (9) and (10):

(11) $$\begin{array}{l} S_{A}\left(x,y,z\right)=\sum_{i}\frac{a}{b_{i}}+\sum_{i}\frac{b_{i}\cos\beta_{i}}{2\pi }\int_{0}^{2\pi }d\theta \int_{0}^{a}{\left(r^{2}+{b_{i}}^{2}-2b_{i}r\sin\beta_{i}\sin\theta \right)}^{-\frac{3}{2}}rdr\\ \;-\sum_{i}\frac{a^{3}\cos\beta_{i}}{2\pi {b_{i}}^{2}}\int_{0}^{2\pi }d\theta \int_{0}^{a}{\left(r^{2}+\frac{a^{4}}{{b_{i}}^{2}}-\frac{2{a^{2}}_{i}r\sin\beta_{i}\sin\theta }{b_{i}}\right)}^{-\frac{3}{2}}rdr \end{array}$$

When actual boundary conditions are considered, the model in the observation cross-section can also be calibrated in a similar way as Equation (8), that is $S_{A,x=0}^{\prime }\left(y,z\right)=-Q_{A,x=0}^{\prime }\left(y,z\right)/q$.

### 3.2. Theoretical Array Signals of an HSESCA

Array signal processing algorithms are included in the definition of the dynamic sensitivity of ESA. In order to find effective array signal processing algorithms for an HSESCA, the characteristics of its array signal shave to be analyzed.

It is assumed that a charged particle moves along the pipeline’s axial direction at a constant velocity [10,32]. In this case, the *x*-coordinates of the particle are denoted with velocity *v* and time *t*, while the *y*-coordinates and *z*-coordinates are regarded as constants. In this way, induced charge on the *i*-th probe is expressed by *Q_i_*(*x*_0_ + *vt*, *y*, *z*), where *x*_0_ is the initial *x*-coordinate. After that, the induced charge is transformed and amplified into voltage according to the amplification coefficient *K* of the signal conditioner (see Figure 4), thus obtaining the out-put signal of the *i*-th sensor unit. That is:

(12) $$u_{i}\left(t\right)=KQ_{i}\left(x_{0}+vt,y,z\right)$$

Then, by combining Equations (4) and (12), one can get:

(13) $$\begin{array}{l} u_{i}\left(t\right)=-\frac{qKa}{b_{i}}-\frac{{qKb}_{i}\cos\beta_{i}}{2\pi }\int_{0}^{2\pi }d\theta \int_{0}^{a}{\left(r^{2}+{b_{i}}^{2}-2b_{i}r\sin\beta_{i}\sin\theta \right)}^{-\frac{3}{2}}rdr\\ \;+\frac{qKa^{3}\cos\beta_{i}}{2\pi {b_{i}}^{2}}\int_{0}^{2\pi }d\theta \int_{0}^{a}{\left(r^{2}+\frac{a^{4}}{{b_{i}}^{2}}-\frac{2{a^{2}}_{i}r\sin\beta_{i}\sin\theta }{b_{i}}\right)}^{-\frac{3}{2}}rdr \end{array}$$

where $b_{i}=\sqrt{{\left(x_{0}+vt\right)}^{2}+{\left(y-y_{i}\right)}^{2}+{\left(z-z_{i}\right)}^{2}}$.

**Figure 5:**
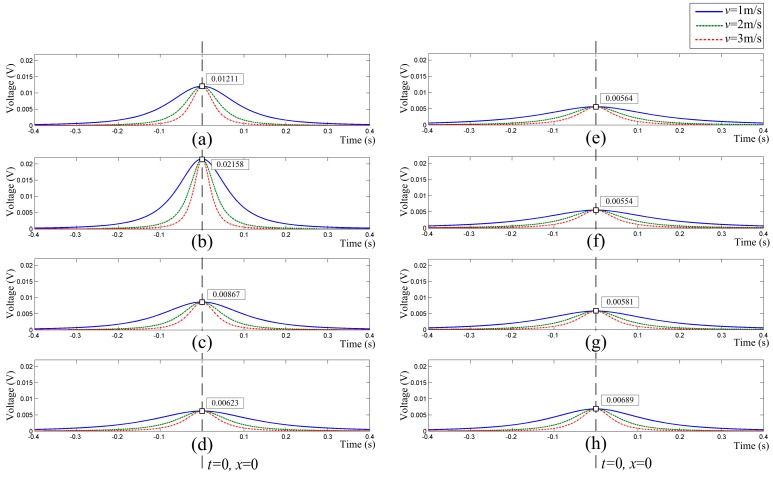
(**a**–**h**) Theoretical array signals of the HSESCA from No. 1–No. 8 sensor units.

In order to provide a visualized view of the array signals, here, we set *K* = −1, *q* = 1C, and the time when the particle reaches the observation cross-section is set as the zero time (i.e., *x*_0_ = 0). The radius of the hemispherical probes is set to 12 mm, and the motion path is set to *y* = 100 mm and *z* = 70 mm (see Figure 3). Besides, the velocities of the charged particle are set to 1 m/s, 2 m/s and 3 m/s, respectively. Then, the array signals of the HSESCA are calculated by using Equation (13), as shown in Figure 5.

This shows that all of the signal peaks *u*_max,*i*_ appear when the charged particle reaches the observation cross-section; when *q* is set as a constant, the *i*-th peak is just determined by the relative position between the motion path and the *i*-th sensor unit, but has nothing to do with the particle’s velocity. Moreover, it is obvious that the peaks will change if the position of the charged particle changes. In a word, different combinations of peaks of the array signals indicate different position information of the charged particle in the observation cross-section.

In order to build a quantitative and direct relationship between a charged particle and the out-put signal peaks of a single electrostatic sensor, the dynamic sensitivity of the electrostatic sensor was proposed by us. It is defined as the absolute value of the out-put signal peak of an electrostatic sensor when a unit point charge passes [9]. As for the *i*-th sensor unit, the relationship is built by its dynamic sensitivity model as:

(14) $$S_{D,i}\left(y,z\right)=\frac{u_{\max,i}}{q}=\frac{KQ_{i,x=0}^{\prime }(y,z)}{q}$$

where $Q_{i,x=0}^{\prime }(y,z)$ is shown as Equation (8).

It is worth noting that in many applications, the monitoring parameters are reflected by the charge quantity of the monitored particles directly or indirectly, such as the particle size, concentration, flow rate, etc. [4,33]. Therefore, it is significant to ensure that the charge quantity of any particle can be accurately monitored no matter where it crosses the observation cross-section. Accordingly, a reasonable way of processing the array signals is as follows: first, obtaining the position information of the monitored particles from the array signals; then, weighting the signal components induced by particles in the less sensitive area according to the position information. In this way, the charge quantity of any particle can be effectively reflected.

### 3.3. Definition and Interpretation of the Dynamic Sensitivity of ESA

The aim of proposing the dynamic sensitivity of ESA is to build a direct relationship between moving charged particles and the monitoring parameters of an ESA, thus reflecting its monitoring accuracy directly. “Dynamic” here has two implications: the first is making a distinction from static sensitivity, that is dynamic sensitivity is proposed for moving charged particles; the second is that the specific expression of the dynamic sensitivity of ESA is changeable according to different array signal processing algorithms. More detailed interpretations are provided by taking intermittent particle monitoring, for example, as follows.

In some applications, the particles to be monitored are intermittent. One representative application is exhaust debris monitoring-based PHM of gas turbines. When faults (e.g., fatigue or carbon deposition) occur on gas path components of gas turbines, some fault-related particles will be produced and charged. Thus, the condition information of the gas path components can be obtained by monitoring the particles [1,6,7,28]. In general, the fault-related particles are intermittent. Moreover, it is considered that larger particles carry greater charge, which indicates more serious faults [1,7]. Therefore, the charge quantity of any fault-related particle should be accurately monitored.

**Figure 6:**
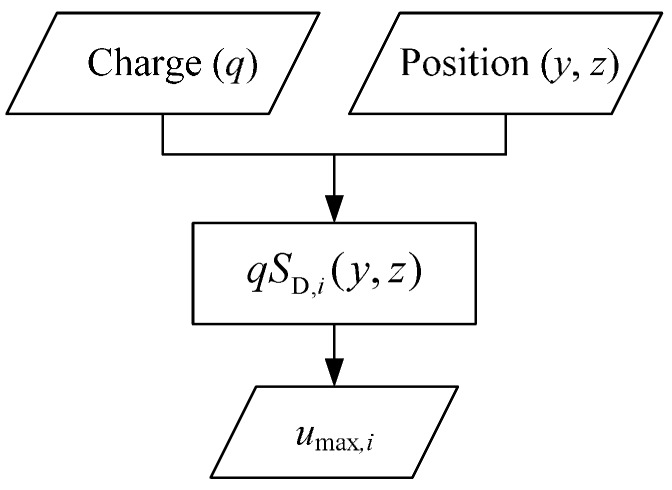
Signal flow diagram of the *i*-th sensor unit of the HSESCA.

As for a single electrostatic sensor, for example the *i*-th sensor unit of the HSESCA, its dynamic sensitivity model builds a relationship between moving charged particles and its out-put voltage signals, as Equation (14). This is expressed by Figure 6.

This means that a charged particle contains two kinds of information, charge quantity *q* and position (*y*, *z*). They codetermine the signal peak *u*_max,*i*_ according to the unit’s dynamic sensitivity model *S*_D,*i*_(*y*, *z*), that is *u*_max,*i*_ = *qS*_D,*i*_(*y*, *z*). However, the relationship is irreversible in practical applications. This is because the position information is difficult to obtain by using a single electrostatic sensor; thus, the value of *S*_D,*i*_(*y*, *z*) is not determined. As a result, the charge quantity *q* cannot be calculated backward for the lack of position information. This is the basic reason for the low monitoring accuracy of a single electrostatic sensor.

**Figure 7:**
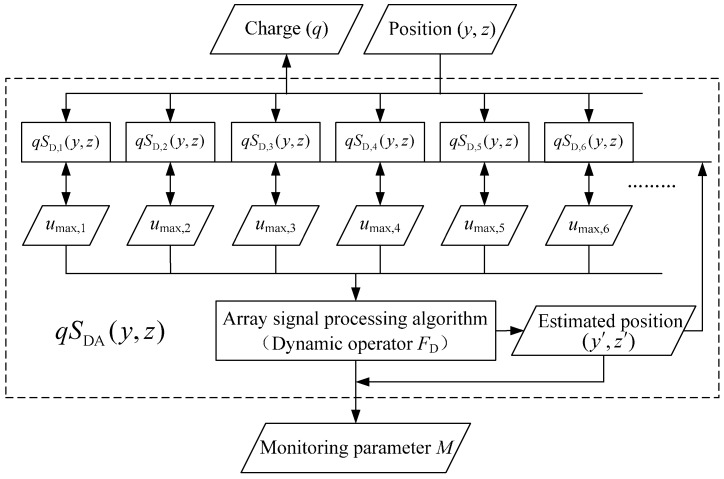
Signal flow diagram of the HSESCA.

It has been mentioned that the array signals of an HSESCA contain the position information of a monitored particle (see Section 3.2), which can be used to improve the monitoring accuracy of the HSESCA. This is expressed by Figure 7.

This means that a charged particle contains two kinds of information, charge quantity *q* and position (*y*, *z*). They codetermine the signal peak *u*_max,*i*_ of each sensor unit according to its dynamic sensitivity model *S*_D,*i*_(*y*, *z*). Then, the peaks are processed by a certain array signal processing algorithm to calculate the monitoring parameter *M*. In this process, the estimated position information (*y*’, *z*’) of the particle is usually obtained and used. Therefore, valves of *S*_D,*i*_(*y*, *z*) are determined by the position information, making it possible to calculate the charge quantity *q* backward. In fact, the monitoring parameters of many applications are just the charge quantity or some derivative values of it. As a result, by using an HSESCA and corresponding array signal processing algorithms, the monitoring accuracy can be improved.

In order to provide a unified and convenient expression for different array signal processing algorithms, dynamic operator *F*_D_ is proposed to denote the array signal processing algorithms. It builds the relationship between the array signals and monitoring parameters of an ESA (see Figure 7).

Further, the dynamic sensitivity of ESA is defined in the observation cross-section as the absolute value of a monitoring parameter when a unit charge passes. As for intermittent particle monitoring, the dynamic sensitivity model of an HSESCA is expressed as:

(15) $$S_{\mathrm{DA}}\left(y,z\right)=\left|\frac{M}{q}\right|=\left|\frac{F_{D}\left[U_{\max}\left(y,z\right)\right]}{q}\right|=\left|\frac{F_{D}\left[qS_{D}\left(y,z\right)\right]}{q}\right|=\left|F_{D}\left[S_{D}\left(y,z\right)\right]\right|$$

where ***U***_max_(*y*, *z*) is the vector of the signal peaks and ***S***_D_(*y*, *z*) is the vector of dynamic sensitivity values of the sensor units. The *q* is reducible because *F*_D_ has to be designed as a linear operator of *q*.

It is seen from Equation (15) that the essence of the dynamic sensitivity of ESA is a recombination of the dynamic sensitivity of the sensor units according to a dynamic operator, which builds a direct relationship between moving charged particles and the system monitor values of an ESA. In other words, the dynamic sensitivity of ESA reflects the monitoring accuracy of an ESA directly by taking array signal processing algorithms into consideration. The concept of dynamic sensitivity has been used by Zhou et al. [34], but the physical meaning is quite different.

## 4. A Component Extraction-Based Array Signal Processing Algorithm for Intermittent Particles

A proper array signal processing algorithm is significant to ensuring the monitoring accuracy of an ESA. As for intermittent particle monitoring (e.g., exhaust debris monitoring-based PHM of gas turbines), it has been mentioned that the accurate charge quantity of any monitored particle is desired; thus, the position information of the particle should be used. In addition, the demand for real-time monitoring should be met to avoid missing momentary faults; thus, the corresponding array signal processing algorithms have to be fast enough.

To meet the conditions above, a component extraction-based array signal processing algorithm is designed. It is based on a simple idea that a monitored particle is located near the sensor units that produce larger signal peaks. According to this idea, a set of common components is extracted from the peaks of the array signals. The components are considered to be generated by a set of imaginary point charges. They have fixed positions, but their imaginary charge quantities vary with the position of the monitored particle. In this way, the position information of the monitored particle can be used according to different charge quantities of the imaginary point charges. It is worth noting that the accurate position of a particle is not needed in the algorithm, but it is made use of by introducing a component extraction-based method. As a result, the algorithm only contains some simple steps, which are convenient for computer processing. More details are provided as follows.

**Figure 8:**
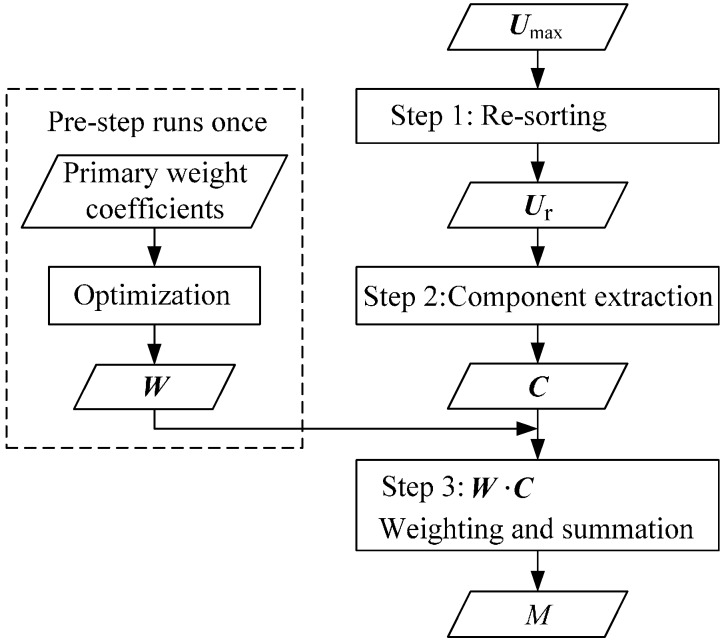
Flow diagram of the component extraction-based array signal processing algorithm.

As shown in Figure 8, three main steps and one pre-step are contained in the component extraction-based array signal processing algorithm. The condition in Figure 4 is taken as an example below, that is to say, the HSESCA contains eight sensor units, and a charged particle moves along a motion path of *y* = 100 mm and *z* = 70 mm.

The first step is re-sorting. When a charged particle passes the observation cross-section, the corresponding signal peaks of the eight sensor units are recorded as a vector ***U***_max_. Then, ***U***_max_ is re-sorted into ***U****_r_* incrementally according to the absolute values of the signal peaks. That is to say, the *i*-th absolute smallest peak is denoted as *u_ri_*; thus, the new vector is ***U****_r_*{*u_r_*_1_, *u_r_*_2_, *u_r_*_3_, *u_r_*_4_, *u_r_*_5_, *u_r_*_6_, *u_r_*_7_, *u_r_*_8_}. For example, the No. 2 probe is the nearest one to the point charge, while the No. 6 probe is the farthest one to the point charge (see Figure 3); thus, according to the fact that nearer probes produce larger signal peaks, one will have *u_r_*_1_ = *u*_max,6_, *u_r_*_8_ = *u*_max,2_.

The second step is component extraction. It is clear that a monitored particle is located near the sensor units that produce larger signal peaks. Thus, if a signal component is only contained in one signal peak, its value implies how close the particle is to the corresponding sensor probe. Analogously, if a signal component is contained in every signal peak, its value implies how close the particle is to the central point. Accordingly, a set of common signal components is extracted from ***U****_r_* by using Equation (16). In detail, the absolute smallest signal peak *u_r_*_1_ is firstly denoted as *c*_1_; it is a common signal component contained in every signal peak. Then, the second absolute smallest peak *u_r_*_2_ is denoted as the sum of *c*_1_ and *c*_2_. It is obvious that *c*_2_ is a common signal component contained in every signal peak, except *u_r_*_1_, and so on; a vector of eight common signal components is extracted from ***U****_r_*, that is ***C***{*c*_1_,*c*_2_, *c*_3_, *c*_4_, *c*_5_, *c*_6_, *c*_7_, *c*_8_}.

(16) $$\left\{ \begin{array}{l} u_{r1}=c_{1}\\ u_{r2}=c_{1}+c_{2}\\ u_{r3}=c_{1}+c_{2}+c_{3}\\ u_{r4}=c_{1}+c_{2}+c_{3}+c_{4}\\ u_{r5}=c_{1}+c_{2}+c_{3}+c_{4}+c_{5}\\ u_{r6}=c_{1}+c_{2}+c_{3}+c_{4}+c_{5}+c_{6}\\ u_{r7}=c_{1}+c_{2}+c_{3}+c_{4}+c_{5}+c_{6}+c_{7}\\ u_{r8}=c_{1}+c_{2}+c_{3}+c_{4}+c_{5}+c_{6}+c_{7}+c_{8} \end{array}\right.$$

**Figure 9:**
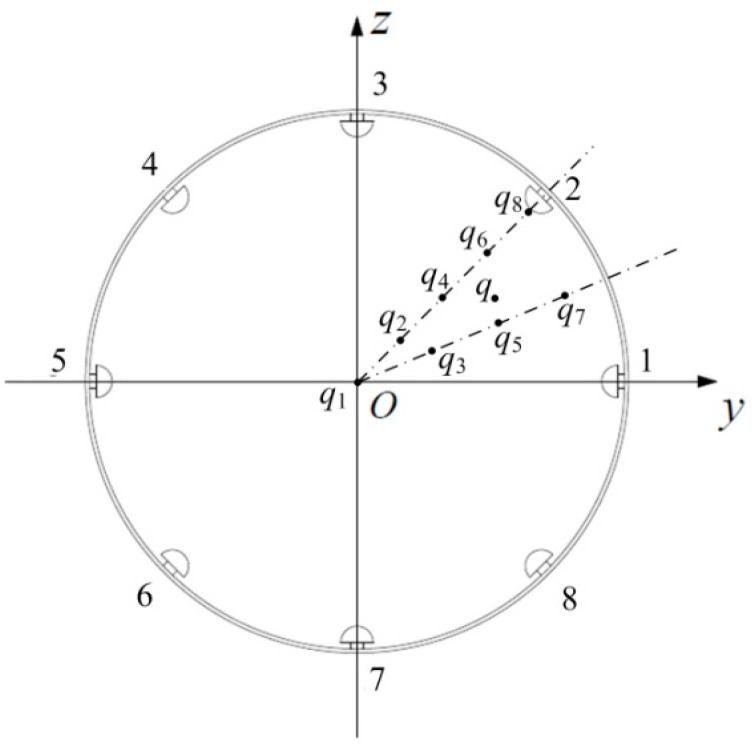
The original charged particle *q* and its imaginary point charges.

The third step is weighting and summation. First of all, the extracted signal components are considered to be generated by a set of imaginary point charges. In detail, based on the symmetry of the circular array and the idea that a charged particle is located near the sensor units that produce larger signal peaks, an imaginary point charge *q*_1_ located in the central point is firstly assumed to generate *c*_1_. Then, because *c*_2_ is contained in every signal peak, except that of the No. 6 sensor unit, an imaginary point charge *q*_2_ is located in the connection line between the origin, and the No. 2 probe is assumed to generate *c*_2_. Analogously, as *c*_3_ is produced by every sensor unit except the No. 5 and No. 6 sensor units, *q*_3_ located in the bisector of the No. 1 and the No. 2 probes is assumed to generate *c*_3_, and so on; eight imaginary point charges are decomposed from the charged particle *q*, as shown in Figure 9. Among them, the point charge *q*_8_ should be quite close to the No. 2 probe, because its relevant signal component is only produced by that probe. The positions of the imaginary point charges are considered to be fixed due to the symmetry of the HSESCA. However, their imaginary charge quantities, which are determined by the values of the extracted components, vary with the position of the monitored particle. In other words, the charge quantities of the imaginary point charges imply the position information of the monitored particle.

Next, the charge quantities of the imaginary point charges are calculated, which can be regarded as a process of weighting the extracted components according to the position information. According to the dynamic sensitivity model of the sensor units as Equation (14), the *i*-th imaginary point charge is expressed as *q_i_* = *c_i_*/*s_i_*, where *s_i_* is the dynamic sensitivity of the No. 2 sensor unit at the position of *q_i_*. By recording:

(17) $$w_{i}=1{/s}_{i}$$

one will have *q_i_* = *w_i_c_i_*, where *w_i_* is just the weight coefficient of *c_i_*.

Finally, the monitoring parameter *M* is obtained by summing up the imaginary point charges:

(18) $$M=\sum_{i}q_{i}=W\cdot C$$

where ***W*** is the vector of the weight coefficients and ***C*** is the vector of the extracted signal components. When *q* is in some symmetric lines of the HSESCA, some components could be zero, but it does not affect the computing process.

***W*** is determined in the pre-step (see Figure 8). Firstly, according to the estimated positions of the imaginary point charges, an initial value of each weight coefficient is set using Equation (17). Then, the initial values are optimized by numerical simulations to make *M* and *q* as close as possible in the whole observation cross-section. In this study, ***W*** was finally determined as {238, 232, 208, 122, 119, 98, 31, 0.9} when *K* = −1. As for a certain HSESCA, the pre-step needs to be done only once, then the weight coefficients are regarded as a priori values in calculating *M*.

According to the definition of the dynamic sensitivity of ESA, the dynamic sensitivity in accordance with the component extraction-based array signal processing algorithm is modeled as:

(19) $$S_{\mathrm{DA}}\left(y,z\right)=\left|\frac{M}{q}\right|=\frac{W\cdot C}{q}$$

This reflects the monitoring accuracy of the HSESCA at different positions in the observation cross-section after adopting the algorithm.

It is seen from the steps that the accurate position of the monitored particle was not obtained. However, the position information was made use of by using the component extraction-based method. In addition, even accurate positions of the imaginary point charges were not necessary. As a result, the proposed array signal processing algorithm only contains some simple steps, making it promising in applications that need fast processing of the array signals.

## 5. Numerical Simulation and Experiment

### 5.1. Simulated Results and Discussion

#### 5.1.1. Simulated Dynamic Sensitivity of the Sensor Units

**Figure 10:**
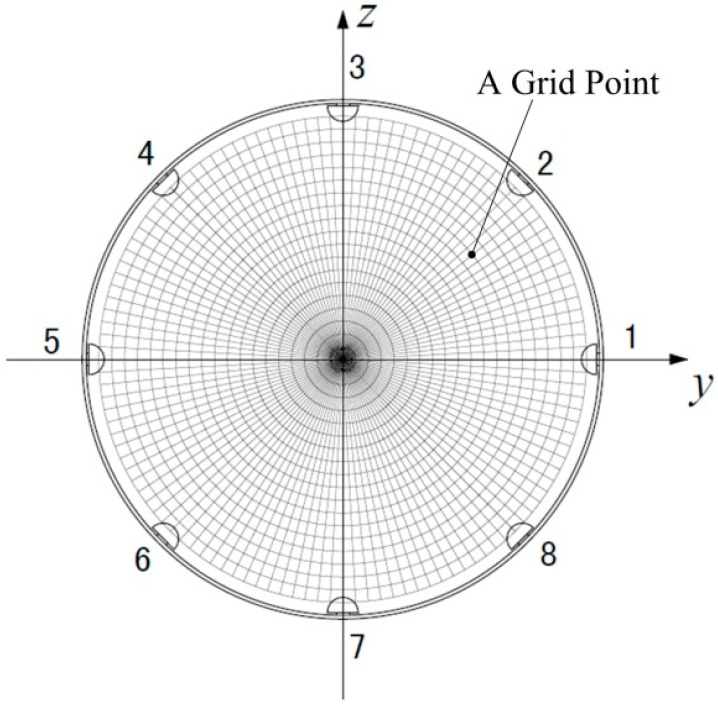
Grid points in the observation cross-section.

As mentioned above, an HSESCA with eight sensor units is installed on a grounded pipeline. The pipeline has an inner radius of 200 mm; the hemispherical probes have a radius of 12 mm with their button faces 2 mm far from the inner wall of the pipeline. For the sake of simplicity, the amplification coefficient of the signal conditioner is set to *K* = −1. Under these conditions, the dynamic sensitivity of the sensor units is calculated by using Equation (14) at the grid points shown in Figure 10. It is seen that the grid points are representative, as they cover almost the whole observation cross-section.

**Figure 11:**
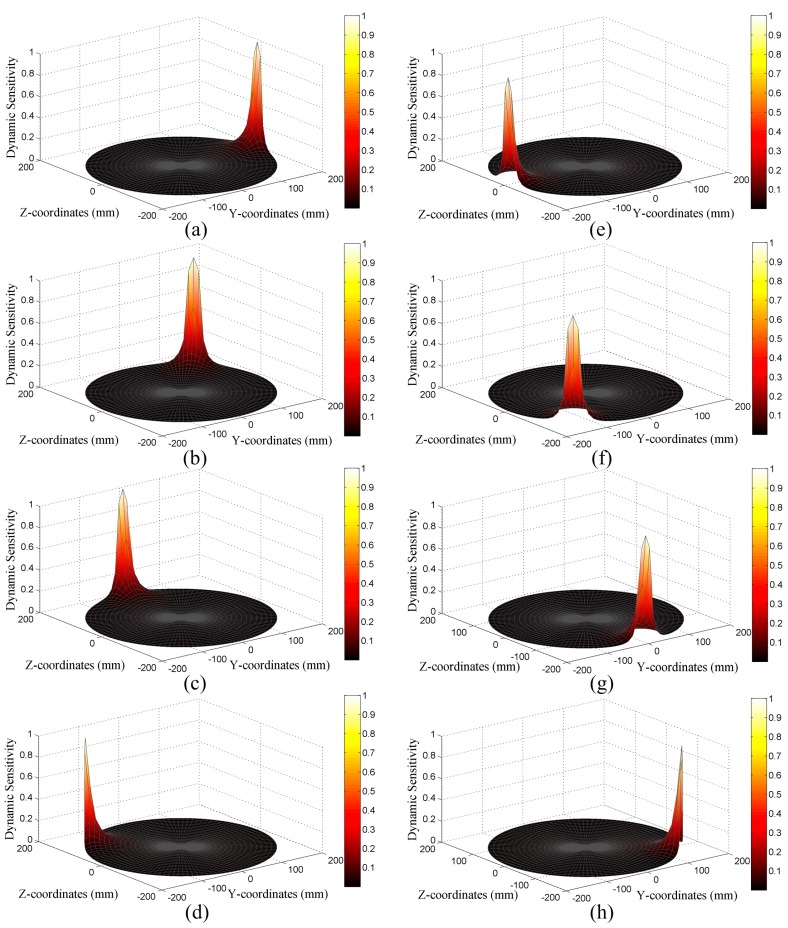
(**a**–**h**) Surface diagrams of dynamic sensitivity of the sensor units from No. 1–No. 8.

**Figure 12:**
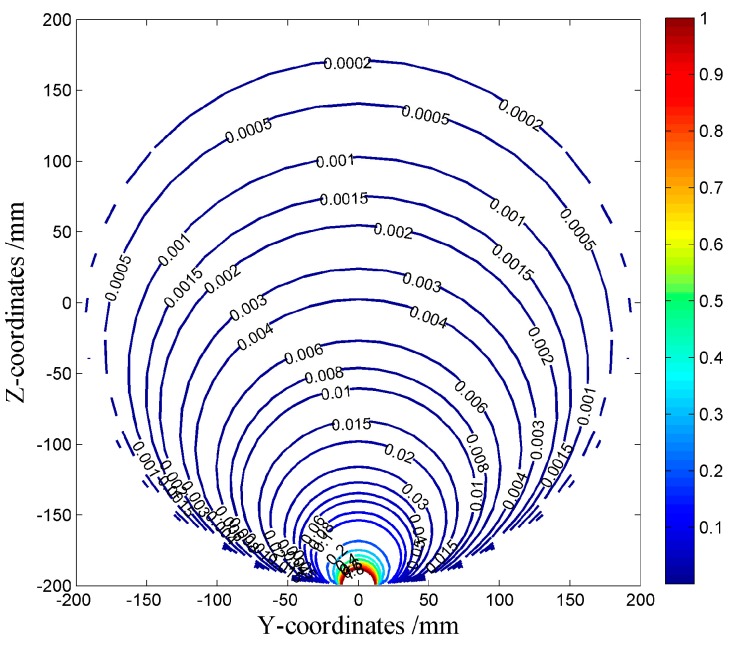
Contour diagram of dynamic sensitivity of the No. 7 sensor unit.

The simulated results are shown by the surface diagrams in Figure 11. It is seen that the sensor units have the same dynamic sensitivity because they are identical in geometry, except that the diagrams rotate 45° in turn, according to the positions of the units. In addition, as for any sensor unit, its dynamic sensitivity reaches the maximum value near the probe surface, and it decreases sharply as the distance to the probe increases. To make a quantitative view, the contour diagram of the No. 7 sensor unit is provided as Figure 12. It shows that the dynamic sensitivity is greater than 0.9 near the probe surface. However, it decreases to below 0.1 just about 30 mm away and decreases to below 0.001 about 150 mm away. In a word, the dynamic sensitivity of the sensor units is inhomogeneous and quite localized, which means that a hemispherical sensor unit can only effectively detect charged particles near its probe. Therefore, it is of practical value to overcome this drawback by using ESAs, thus improving the monitoring accuracy.

#### 5.1.2. Simulated Static Sensitivity of the HSESCA

**Figure 13:**
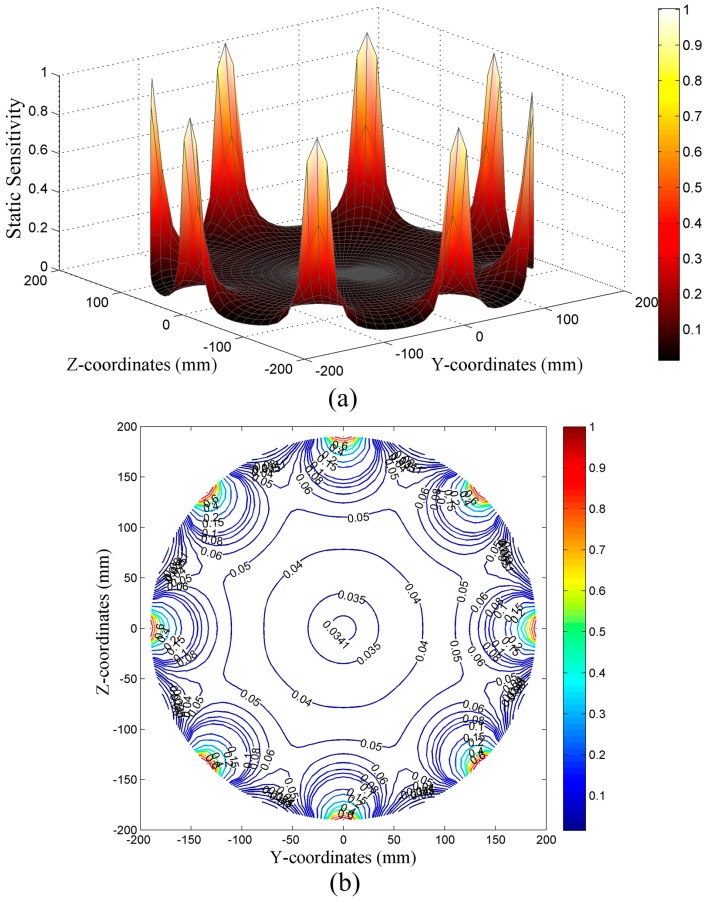
(**a**) Surface and (**b**) contour diagrams of the static sensitivity of the HSESCA.

The static sensitivity of the HSESCA in the observation cross-section is calculated by using Equation (11) and the calibration method. The results are shown in Figure 13.

It is seen from Figure 13 that, in the observation cross-section, the static sensitivity of the HSESCA distributes symmetrically in accordance with the distribution of the probes. In addition, just like the dynamic sensitivity of a certain sensor unit, the static sensitivity of the HSESCA reaches the maximum value greater than 0.9 near the probes, and it decreases sharply as distance to the probes increases. In most of the central area, the values of static sensitivity are below 0.05. That is to say, the static sensitivity of the HSESCA is inhomogeneous and quite localized, which means that an HSESCA will only effectively detect charged particles near the probes if just using the summation of the induced charges to reflect the charge quantity of a monitored particle. Definitely, the static sensitivity in the center region can be improved by increasing the size and number of probes. However, this will cause problems in installation and increase the disturbance to the flow. Therefore, it is advisable to ensure the monitoring accuracy in the center region by using appropriate array signal processing algorithms.

#### 5.1.3. Simulated Dynamic Sensitivity of the HSESCA

**Figure 14:**
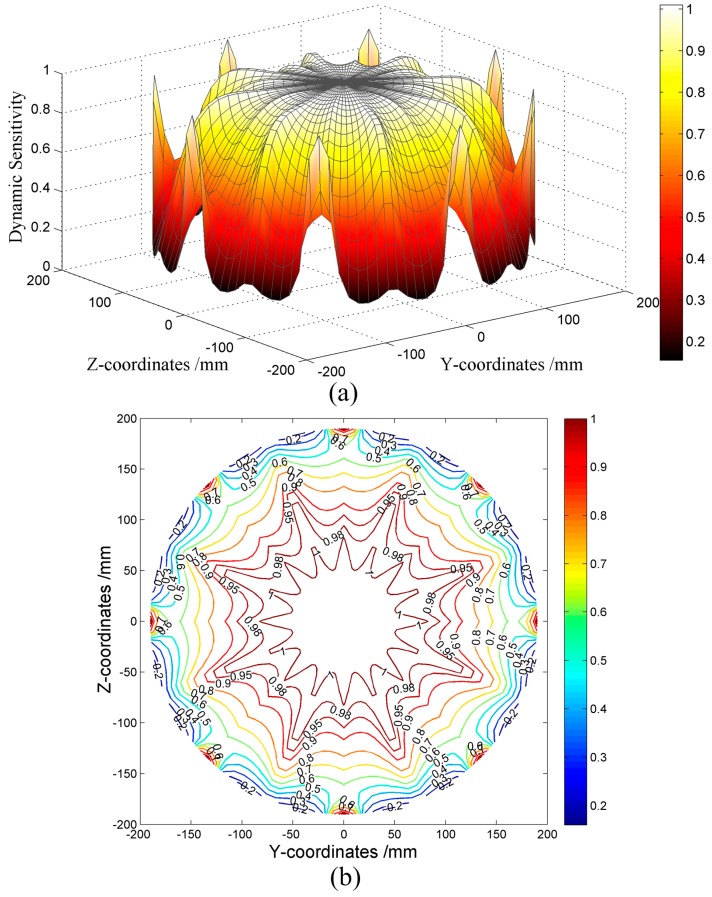
(**a**) Surface and (**b**) contour diagrams of the dynamic sensitivity of the HSESCA.

For the sake of simplicity, here, we set the amplification coefficient *K* = −1. Then, by placing a point charge *q* = 1 C in turn at each grid point shown in Figure 10, the corresponding signal peaks of all of the sensor units are calculated using Equation (14). Next, they are processed by the component extraction-based array signal processing algorithm (see Section 4) to get the monitoring parameter value corresponding to each grid point. In this way, the values of dynamic sensitivity at all of the grid points are obtained. The results are shown in Figure 14.

It is seen from Figure 14 that the dynamic sensitivity of the HSESCA distributes symmetrically in accordance with the distribution of the probes. In addition, it is obvious that the dynamic sensitivity has relatively high values, not only near the probes, but also in most of the area of the observation cross-section. By making a comparison between Figure 13 and Figure 14, it is shown that the dynamic sensitivity and the static sensitivity have close values near the probes and the inner wall of the pipeline. However, the mean value of the static sensitivity is only about 0.04 in most of the central area, while that of the dynamic sensitivity reaches about 0.95. The sensitivity represents charge measurement accuracy, that is to say the closer to one the sensitivity is, the higher the accuracy is. Therefore, in the case of intermittent particle monitoring, the monitoring ability gets enhanced by 23 times in most of the central area because of using the component extraction-based array signal processing algorithm, which implies significant improvements in sensitivity homogeneity and monitoring accuracy of the HSESCA. Furthermore, it shows that there still distribute relatively low values near the inner wall of the pipeline, except where the probes are located. This is unavoidably caused by the influence of the pipeline on the electrostatic field. However, using a strategy to optimize the number of sensor units combined with optimizations on the proposed algorithm is likely to alleviate this drawback, which deserves further studies.

### 5.2. Experimental Results and Discussion

#### 5.2.1. Experiment Apparatus

**Figure 15:**
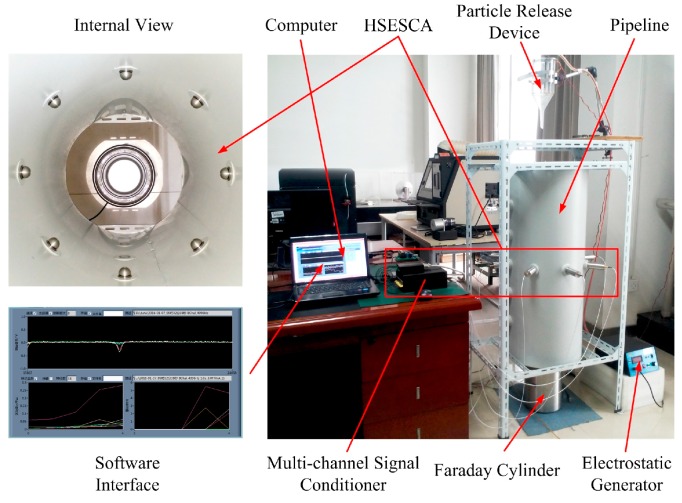
Structure of the HSESCA experiment apparatus.

Experimental results were obtained by using an eight-channel HSESCA experiment apparatus. As shown in Figure 15, the experiment apparatus is mainly composed of an electrostatic generator, a particle release device, a pipeline, an HSESCA, a Faraday cylinder and a computer. The electrostatic generator produces ionized air in the particle release device by high voltage, which makes released particles charged. An HSESCA that has eight sensor units is installed in the midsection of the grounded pipeline. It produces array signals when a charged particle falls through the pipeline. Then, the signals are processed by using specially-designed software integrated in the computer. The Faraday cylinder connected with a charge meter is placed below the pipeline. A charged particle will finally fall into the Faraday cylinder; thus, the charge quantity of the particle is read from the charge meter with a measurement resolution of 1 pC. Steel balls with a diameter of 2 mm are used as the experimental particles. To create a consistent condition with the numerical simulation, the pipeline has an inner radius of 200 mm; the hemispherical probes have a radius of 12 mm with their button faces 2 mm far from the inner wall of the pipeline.

**Figure 16:**
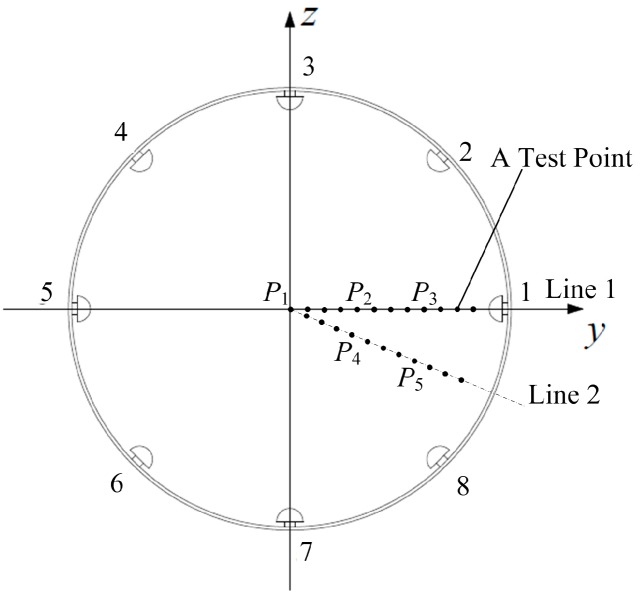
Test points set in the experiment.

In the experiment, the particles were released at different test points, as shown in Figure 16. The points were set along two typical lines in the observation cross-section. Line 1 is the axis of the No. 1 sensor unit, and Line 2 is the bisector of the No. 1 and the No. 8 sensor units. Each line contains 12 test points, which are uniformly spaced by 15 mm. In order to reduce the position error of each release and the measurement error of the charge quantity, 10 tests were done at each point. Then, the mean valves of the measured signal peaks and those of the charge quantities were further used to calculate the static sensitivity and the dynamic sensitivity of the HSESCA.

#### 5.2.2. Array Signals of the HSESCA

**Figure 17:**
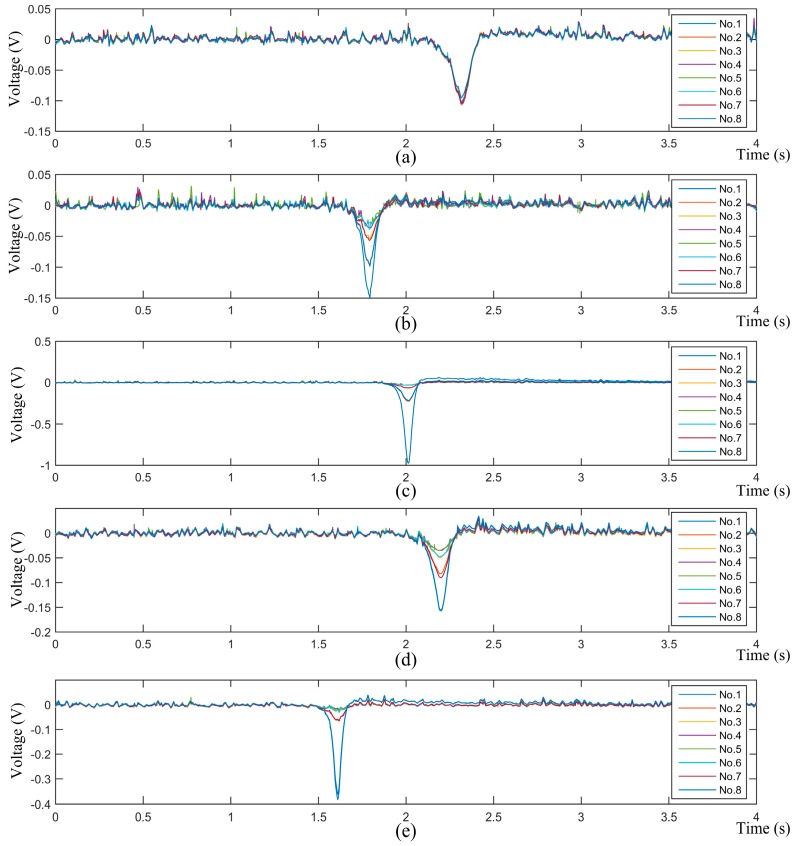
Array signals of the HSESCA. (**a**) Released at *P*_1_ with −32 pC charge; (**b**) released at *P*_2_ with −20 pC charge; (**c**) released at *P*_3_ with −46 pC charge; (**d**) released at *P*_4_ with −25 pC charge; (**e**) released at *P*_5_ with −29 pC charge.

Five sets of typical array signals with a sample frequency of 4096 Hz are shown in Figure 17. They were acquired when particles were released at *P*_1_, *P*_2_, *P*_3_, *P*_4_ and *P*_5_, respectively (see Figure 16). Among them, *P*_1_ is the origin; *P*_2_ and *P*_4_ are 60 mm away from the origin; *P*_3_ and *P*_5_ are 120 mm away from the origin. Besides, the charge quantities of the particles were measured to be −32 pC, −20 pC, −46 pC, −25 pC and −29 pC, respectively.

Figure 17 shows that the measured signals are similar in shape as those obtained by numerical simulations (see Figure 5), except the sign symbol. The measured ones are minus because of the minus charge quantities of the particles. Besides, a signal variation trend versus the positions in Line 1 can be observed from Figure 17a–c. In detail, the signals of all of the sensor units are almost coincident at *P*_1_, which is explained by the same distance from *P*_1_ to every probe. However, with the release position moving from *P*_1_ to *P*_3_, the signal peak of the No. 1 sensor unit gets larger and larger. Conversely, other signal peaks decrease with respect to that of the No. 1 sensor unit; among them, that of the No. 5 sensor unit decreases the most. In addition, two symmetric sensor units about Line 1 have the same decrease scale in their signal peaks. A similar trend can also be observed when the release position moves from *P*_1_ to *P*_5_ along Line 2. This is because a charged particle induces greater charge on closer probes, and vice versa. In a word, the measured array signals have validated the theoretical ones in form. Moreover, it is validated that different combinations of peaks of the array signals imply different position information of the particles. This agrees with the theoretical analysis and is the precondition of the proposed array signal processing algorithm.

**Figure 18:**
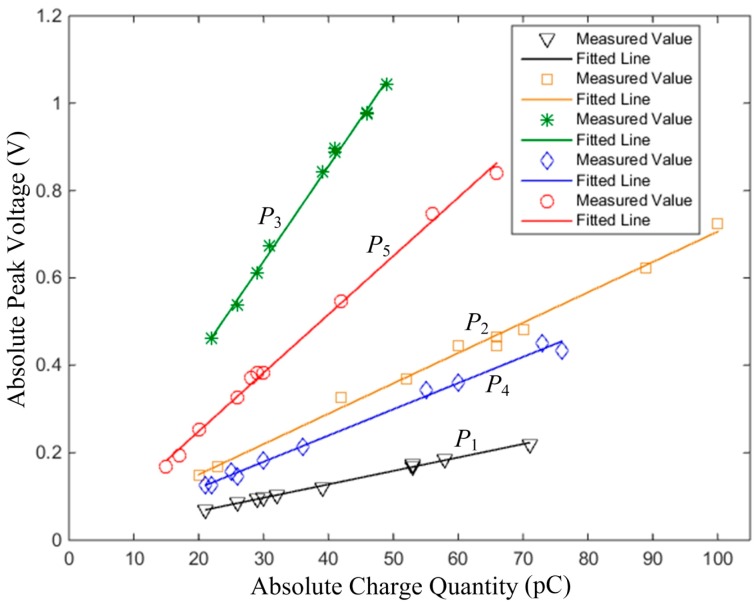
Relationships between the absolute charge quantities and the absolute peak voltages of the No. 1 sensor unit.

Ten tests were done at the release positions of *P*_1_, *P*_2_, *P*_3_, *P*_4_ and *P*_5_, respectively. The absolute charge quantities of the particles and the absolute signal peaks of the No. 1 sensor unit were measured. Their relationships are shown in Figure 18.

This shows an obvious proportional relationship between the absolute charge quantities and the absolute peak voltages at each position. The different slopes indicate different values of dynamic sensitivity with respect to different positions, and greater slopes represent greater values of dynamic sensitivity. The errors are acceptable and can be explained by the position error of each release and the measurement error of the charge quantity. As a result, the proportional characteristic of the signal conditioner is validated.

#### 5.2.3. Static Sensitivity Test of the HSESCA

**Figure 19:**
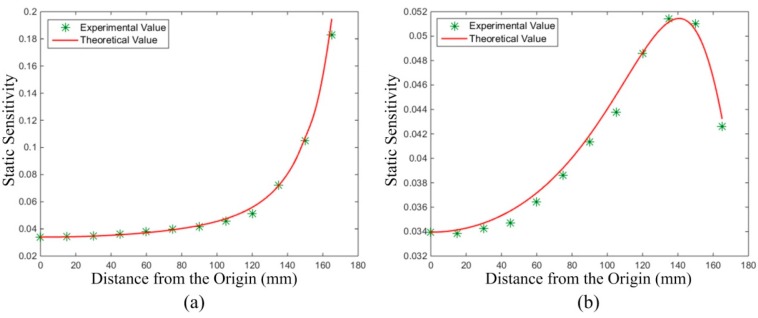
Experimental and theoretical values of static sensitivity (**a**) in Line 1 and (**b**) in Line 2.

To make a comparison between the theoretical and experimental results of the static sensitivity of the HSESCA, the theoretical values in Line 1 and Line 2 were firstly calculated by using Equation (11) and the calibration method when *K* = −1. Then, according to the definition of static sensitivity as Equation (10), the experimental value at each test point was obtained by dividing the summation of all of the signal peaks by the charge quantity of the corresponding particle, followed by a scaling to suppose *K* = −1. The results are shown in Figure 19, where the curves represent the theoretical values and the points represent the experimental values.

It can be seen that the experimental values show fine consistencies with the theoretical ones. Further analysis shows that the mean absolute value of relative error from all of the test points in Line 1 is 2.2544%, and that in Line 2 is 1.3516%. The errors are acceptable and can be explained by the errors in controlling the particles’ falling positions and measuring their charge quantities. Therefore, the sensing model of the HSESCA, as well as that of the sensor units are validated.

#### 5.2.4. Dynamic Sensitivity Test of the HSESCA

**Figure 20:**
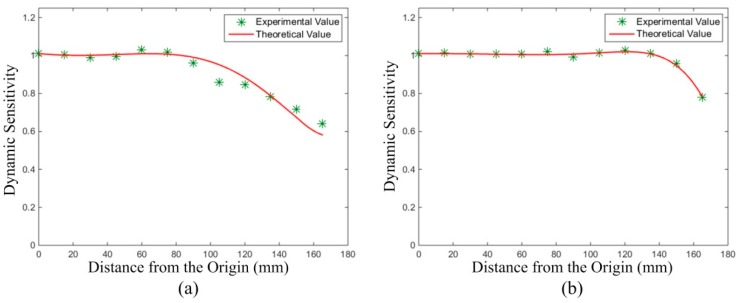
Experimental and theoretical values of dynamic sensitivity (**a**) in Line 1 and (**b**) in Line 2.

Theoretical and experimental results of the dynamic sensitivity of the HSESCA were also compared. When the amplification coefficient was set to *K* = −1, the theoretical values in Line 1 and Line 2 were firstly calculated (see Section 5.1.3). After that, the measured signal peaks were processed by the component extraction-based array signal processing algorithm to obtain the experimental values. In detail, the peaks were decomposed, weighted and summated to obtain the values of the monitoring parameter. Then, the corresponding values of dynamic sensitivity are calculated by using Equation (19), followed by a scaling to suppose *K* = −1. The results are shown in Figure 20, where the curves represent the theoretical values and the points represent the experimental values.

It is observed that the experimental values match well with the theoretical ones. Further analysis shows that the mean absolute value of relative error from all of the test points in Line 1 is 3.3233%, and that in Line 2 is 0.5936%. Just as the results in the static sensitivity test, the errors here are acceptable and can be explained by the errors in controlling the particles’ falling positions and measuring their charge quantities. In addition, by making a comparison between Figure 19 and Figure 20, it is seen that the values of dynamic sensitivity are larger than 0.8 in most of the section of Line 1 and Line 2, but those of the static sensitivity are almost below 0.1. This demonstrates that the monitoring accuracy of the HSESCA for intermittent particles has been improved significantly by using the component extraction-based signal processing algorithm, which agrees with the theoretical analysis above. Therefore, the proposed algorithm is proven to be effective in actual measurements. More details can be found in Section 5.1.2 and Section 5.1.3.

## 6. Conclusions

By taking the processing of the array signals into consideration, the dynamic sensitivity of ESA has been defined to describe the sensitivity characteristics of ESAs in a systemic perspective. It builds a direct relationship between the monitored particles and the monitoring parameters of a whole ESA, thus reflecting the monitoring accuracy directly. An HSESCA along with its application in intermittent particle monitoring has been taken as an example. Its dynamic sensitivity in accordance with a proposed component extraction-based array signal processing algorithm has been analyzed quantitatively. Relevant numerical simulations have been made, and experimental validations have been carried out on an eight-channel HSESCA experiment apparatus. Detailed results have been provided, compared and discussed, which are summarized as follows:

1. The experimental results match well with the theoretical ones, which has validated the accuracy of the theoretical models and effectiveness of the corresponding methods.
2. Compared with static sensitivity, the dynamic sensitivity of the HSESCA has much greater values in most of the observation cross-section. This demonstrates that the component extraction-based array signal processing algorithm is effective at overcoming the defect of inhomogeneous and localized static sensitivity, thus making a significant improvement on the monitoring accuracy for intermittent particles.
3. There still exist relatively less sensitive zones near the inner wall of the pipeline after adopting the proposed algorithm. This is caused by the influence of the pipeline on the electrostatic field. A strategy to optimize the number of sensor units combined with optimizations on the proposed algorithm is likely to alleviate the drawback, which deserves further studies.
4. Ill-conditioning and noise may impact the results of the proposed array signal processing algorithm. This is of great significance, to be discussed in further studies.

## Abbreviations

EMS	Electrostatic monitoring system
ESA	Electrostatic sensor array
HSESCA	Hemisphere-shaped electrostatic sensors’ circular array
PHM	Prognostics and health management
